# Food Consumption Patterns and Nutrient Intakes of Children and Adolescents in the Eastern Mediterranean Region: A Call for Policy Action

**DOI:** 10.3390/nu12113345

**Published:** 2020-10-30

**Authors:** Ayoub Al-Jawaldeh, Mandy Taktouk, Lara Nasreddine

**Affiliations:** 1 World Health Organization (WHO), Regional Office for the Eastern Mediterranean (EMRO), Cairo 7608, Egypt; aljawaldeha@who.int; 2 Nutrition and Food Sciences Department, Faculty of Agriculture and Food Sciences, American University of Beirut, Beirut 11-0236, Lebanon; mrt07@mail.aub.edu

**Keywords:** food consumption patterns, dietary intakes, macronutrients, micronutrients, children, adolescents, Eastern Mediterranean Region, review

## Abstract

The Eastern Mediterranean Region (EMR) has witnessed significant social and economic changes that may have influenced the diet of children and adolescents, and increased the risk for obesity and malnutrition in this age group. This review aims to characterize and assess food consumption patterns and nutrient intakes amongst school-aged children (5–10 years) and adolescents (10–19 years) in countries of the EMR. Electronic databases (MedLine, PubMed, Scopus, and Google Scholar) were searched for relevant articles published between 2005 and 2020; international organizations and governmental websites were also searched. Available studies documented low intakes of fruits, vegetables and fiber, inadequate consumption of water, milk and dairy products, coupled with high intakes of fat, saturated fat, and sugar sweetened beverages, as well as a frequent consumption of energy-dense, nutrient poor foods such as sweet and savory snacks. Micronutrient inadequacies were also observed, particularly for calcium, iron, zinc and vitamins A, D, C, and folate. Acknowledging the impact that nutrition may have on building societies and transforming the lives of children, adolescents and their families, there is a crucial need for a food system approach in developing and implementing national and regional policies and interventions aimed at improving the diet of children and adolescents.

## 1. Introduction

The health and well-being of children and adolescents are essential prerequisites for achieving the Sustainable Development Goals (SDGs), particularly those focusing on poverty, health security, education and the reduction of inequalities [1]. The World Health Organization (WHO) acknowledged the importance of adequate nutrition to “enable children and adolescents to enjoy good health while playing a full role in contributing to transformative change and sustainable development”, in alignment with the SDGs [2]. Good nutrition during childhood and adolescence is in fact indispensable for growth and development, health and well-being, and the prevention of obesity and several chronic diseases [3].

Unhealthy diets in childhood and adolescence are associated with immediate as well as long-term health impacts. In the short term, inadequate dietary intakes of energy, protein, or certain micronutrients will result in slower growth rates, delayed sexual maturation, lower reserves of micronutrients, and inadequate bone mass [4]. Dietary intakes of children and adolescents may also affect their risk of developing a number of health problems, such as iron deficiency and dental caries, while also lowering their resistance to infectious diseases and adversely affecting their ability to function at peak mental and physical capacity [3]. Poor diets in these critical periods of the life course are also linked with pediatric obesity and its related metabolic abnormalities, such as high blood pressure, type 2 diabetes (T2D), metabolic syndrome, sleep disturbances, orthopedic problems, and psychosocial problems [5,6,7,8], which all tend to track into adulthood [9].

Dietary practices of children and adolescents may also carry long-term health ramifications, increasing the risk for several non-communicable diseases (NCDs), and contributing significantly to the burden of preventable diseases and premature deaths [3]. In the Eastern Mediterranean region (EMR), which has witnessed over the past few decades important social, economic, and political changes [10], three of the ten leading causes of death are related to dietary factors, including ischemic heart disease, strokes, and diabetes. Urbanization, technological development, and modernization, have in fact instigated significant demographic and epidemiologic changes in most countries of the region, with parallel shifts in diet, physical activity, and body composition [11]. These shifts represent the basis of the multidimensional phenomenon of the nutrition transition, which is characterized by increases in the intakes of energy, fat, added sugars, and salt [11,12]. Some states in the region are classified as countries in advanced nutrition transition, such as the Gulf Cooperation Council (GCC) countries, the Islamic Republic of Iran, and Tunisia, while others are classified in early nutrition transition, such as Jordan, Lebanon, Egypt, Libya, and Morocco [13]. In contrast, political turmoil and economic challenges have adversely impacted the availability of food in some EMR countries such as Iraq, Pakistan, occupied Palestinian territory, and Yemen, while some states are categorized as countries in emergency and humanitarian crisis, such as Afghanistan, Somalia, and Sudan [13,14].

Amidst the threat of transitioning to high-energy, nutrient-poor diets and the parallel hazard of food insecurity, children and adolescents may be amongst the most vulnerable population groups to the ongoing societal, lifestyle, and dietary changes in countries of the region [15]. Available evidence indicates that the region harbors one of the highest rates of pediatric and adolescent obesity worldwide, while the burden of undernutrition and micronutrient deficiencies persists in many of its countries [16]. Overcoming pediatric and adolescent malnutrition in all of its forms (overnutrition, undernourishment, and micronutrient deficiencies) entails the development of evidence-based interventions and the design of related health policies to ensure the availability of and access to healthy diets. Effective planning for such interventions should be guided by accurate, up-to date and comprehensive data on food consumption patterns and nutrient intakes. The objective of this review is to characterize and assess food consumption patterns and nutrient intakes amongst school-aged children (5–10 years) and adolescents (10–19 years) in countries of the EMR. Findings from this review will characterize food consumption patterns amongst children and adolescents in the EMR and identify prevalent nutrient excesses or inadequacies. It will also contribute to the prioritization of research to address current gaps in knowledge and inform policies and interventions aimed at developing healthy eating habits in these critical periods of the lifecycle.

## 2. Approach

The literature search covered the EMR, which according to the WHO, includes 21 countries that comprise Afghanistan, Bahrain, Djibouti, Egypt, Iran (Islamic Republic of), Iraq, Jordan, Kuwait, Lebanon, Libyan Arab Jamahiriya, Morocco, Oman, Pakistan, Qatar, Saudi-Arabia, Somalia, Sudan, Syrian Arab Republic, Tunisia, United Arab Emirates (UAE), and Yemen [17].

Dietary intake data including food group, energy, macronutrient, and micronutrient intakes were evaluated and compared with reference intake values and/or guidelines (when available). A comprehensive literature review was conducted, including individual studies and review articles published between 2005 and 2020, which reported on dietary intakes in children and adolescents aged 5–19 years in any country of the EMR. Electronic databases (MedLine, PubMed, Scopus, and Google Scholar) were searched between 15 July 2020, and 15 August 2020. The search was restricted to the English, French, and Arabic languages, and the key terms used in the search strategy were as follows: EMR countries and/or each country alone AND “Diet” OR “Dietary” OR “Nutritional” OR “Nutrient” AND “Intake” OR “Consumption” AND “Children” OR “Child” OR “Adolescent”. In addition, for the dietary intake, a narrower search was performed while including the following key terms: “Energy”, “Macronutrient”, “Carbohydrate”, “Fat”, “Saturated Fat”, “trans-fat” “Protein”, “Fiber”, “Sugar”, “Meats”, “Milk”, OR “Dairy”, “Fruits”, “Vegetables”, “Candy”, OR “Candies”, OR “Sweets”, “Chips”, “Water”, “Sugar Sweetened Beverage”, “Juice”. A parallel search strategy was also adopted for micronutrient intake (including iron, iodine, zinc, copper, calcium, sodium, thiamin, riboflavin, folate, vitamin B12, vitamin A, vitamin D, and vitamin C). The reference lists of the specific studies were also reviewed to identify additional data sources. Studies were retained if they reported on children aged 5 to 18 years; studies reporting on under-five children were excluded. The global school-based student health survey (GSHS) database [18] was also reviewed to obtain data on the consumption of fruits, vegetables, and carbonated beverages.

Data was presented separately for school-aged children (5–10 years) and adolescents (10–19 years). Food groups were classified based on the categories proposed by Keats et al. [19] in their systematic review of dietary intakes amongst adolescent girls in low and middle income countries. Accordingly, the following food categories were adopted: (1) fruits; (2) vegetables; (3) pulses (beans, peas, lentils); (4) grains; (5) dairy; (6) meat, poultry, and fish; (7) fast foods; (8) sweet snacks; (9) salty and fried snacks, and (10) sugar-sweetened beverages (SSBs). Dietary intakes were compared, when possible, with the WHO recommendations. The recommendations of the WHO for a healthy diet include the consumption of at least 400 g, or 5 portions, of fruits and vegetables/day to reduce the risk of NCDs and ensure an adequate daily intake of dietary fiber (>25 g); reducing sodium intake to less than 2 g/day (5 g of salt), total fat intake to less than 30% of energy intake (EI), reducing saturated fat (SFA) intake to less than 10% EI, and trans fat (TFA) to less than 1% EI [20], and replacing them with unsaturated fats including polyunsaturated fats (PUFAs) (6–10% EI), n-6 PUFAs (5–8% EI), and n-3 PUFAs (1–2% EI); consuming protein in the range of 10–15% EI, carbohydrates 55–75% EI, and free sugars (FS) less than 10% EI.

## 3. Results

### 3.1. Food Consumption Patterns Amongst Children and Adolescents in the EMR

#### 3.1.1. School-Aged Children

A striking scarcity is noticed with respect to studies investigating food consumption patterns amongst school-aged children in the EMR. Many of the available studies were conducted with the aim of investigating the relationship between dietary factors and health outcome, such as dental caries [21,22,23], overweight [24,25], elevated blood pressure [26], or anemia [27], in small samples of children. The majority of available studies have reported on whether the child consumes a certain food group (Yes/No) or on only the frequency of intake (per day or per week), which limits the interpretability of the findings.

In the Levant, a national study conducted in Lebanon showed that fast food alone contributed to around 11.3% of daily EI, and this was coupled with a high intake of sweets and SSBs, which provided 10.8% and 6.5% EI, respectively [24]. In the GCC, a study conducted amongst children aged six years and above in the 11 regions of Bahrain (*n* = 496) reported that only half of the children reported daily consumption of milk and its products and one fourth reported daily consumption of fruits and vegetables [28]. In contrast, daily consumption of soft drinks was reported by 50% of the children and daily consumption of sweets and snacks was reported by 64% of girls and 47% of boys [28]. A study conducted in all the seven emirates of the UAE showed that amongst 6–8 year old children, more than 90% did not meet the MyPyramid recommendations for vegetables and milk/dairy products, 72–89% did not meet the recommendations for fruits, 49–68% did not meet the recommendations for grains, and 64–75% did not meet the recommendations for meat and beans [29]. In the Kingdom of Saudi-Arabia (KSA), a study conducted amongst 7–12 year old children in Al-Baha city indicated that, 69% and 71% of children did not report daily consumption of fruits and vegetables, respectively, and that only 0.9% met the recommended intake levels of fruits and vegetables [30]. This study also showed that 32% of children did not consume milk/dairy products on a daily basis, with only 1.9% adhering to dairy intake recommendations [30]. In Qatar, a study investigating snack consumption amongst 9–10 year old students in Doha showed that the most commonly consumed snacks in the study sample included fruit drinks (consumed by 98.8% of students), and a high percentage of children reported the consumption of potato chips (81.5%), candy and chocolates (41.7%), and pizzas and pies (39.8%), while the least consumed snacks were milk (37.1%) and nuts (0.8%) [31].

Studies conducted in Iran reported a relatively high consumption of fruits and vegetables amongst school-aged children, in the range of 400 g/day [20,32], with more than 60% of children being adherent to the WHO recommendations [33]. In contrast, the consumption of milk and dairy products was reported to be low (0.8 servings/day) [34]. A high consumption of sweet snacks (two servings/day) [32], fats and oils (6.2 servings/day) [34], and salty snacks (1.3–1.4 servings/day) [32] was also noted. The average consumption of grains met the recommendations (7.4 servings/day) [34], but approximately 60% of children reported to consume grains in their refined form [35].

#### 3.1.2. Adolescents

**Figure 1:**
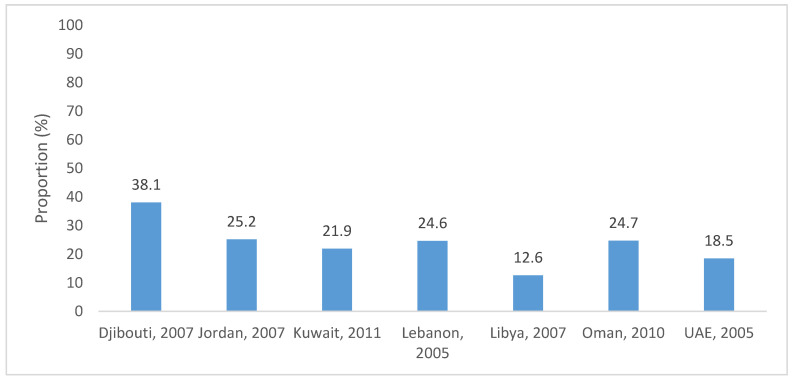
Proportion of students (aged 13–15 years) who had fruits and vegetables at least five times/day during the 30 days preceding the survey, based on the global school-based student health survey (GSHS) database [18].

**Figure 2:**
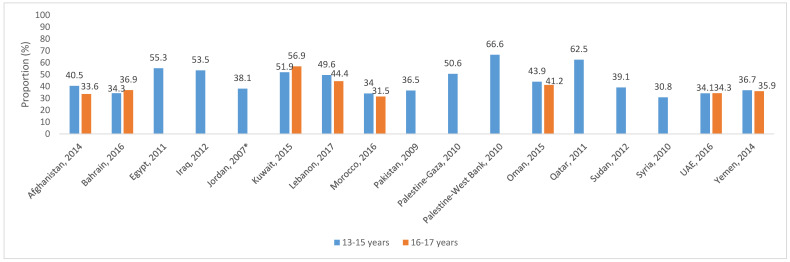
Proportion of students (aged 13–17 years) who drank carbonated soft drinks once or more times per day during the 30 days preceding the survey, based on the global school-based student health survey (GSHS) database [18]. *: This data represents the proportion of students (13–15 years) who drank carbonated soft drinks twice or more times per day during the 30 days preceding the survey in Jordan.

The GSHS database provides country-specific information on the consumption of fruits, vegetables, and carbonated beverages amongst adolescents aged 13–17 years [18]. Figure 1 summarizes data available from EMR countries. Amongst 13–15 year old adolescents, the percentage of students who reported the consumption of fruits and vegetables at least five times/day during the month preceding the survey was low, ranging between 12.6% in Libya and 38.1% in Djibouti. In contrast, higher proportions of adolescents reported the consumption of carbonated soft drinks once or more times/day. These proportions ranged between 30.8% and 66.6% amongst 13–15 year old adolescents and between 31.5% and 56.9% in those aged 16 to 17 year old (Figure 2).

Research studies have also documented suboptimal dietary practices amongst adolescents in the EMR. Table 1, shows that the proportions of adolescents reporting daily intake of fruits ranged between 11% and 33.5% in most countries, except for studies conducted in Iraq [36] and Palestine [37] where higher estimates were reported. The proportions of adolescents reporting daily consumption of vegetables ranged between 20% and 43% in countries like Bahrain, Jordan, Kuwait, Morocco, Qatar, and KSA, while higher estimates were reported from Egypt (78.2%), Iraq (46–62%), Palestine (73%), and Sudan (70%) (Table 1). In Lebanon, a study investigating the diets of adolescents from contrasting socioeconomic backgrounds (*n* = 209, aged 17–19 years) [38] documented low intakes of vegetables, ranging between 1.4 and 1.9 servings/day, while fruits intake was reported as adequate. Studies conducted in Oman showed that, amongst adolescents, 52–57% consumed less than three servings of vegetables/day [39], and more than a third of adolescents consumed less than two servings of fruits/day [39]. In the UAE, Makansi et al. (2018) indicated that only 28% of adolescents from grades 10–12 (*n* = 620) met the recommended daily fruit and vegetable intake [40]. In a study conducted amongst 12–16 year old adolescents in Shiraz (Iran), the intake of fruits and vegetables together was estimated at 3.27 servings/day [41].

Food consumption patterns amongst adolescents in the region are also characterized by inadequate intakes of dairy: the proportions reporting daily intake did not exceed a third of adolescents in EMR countries, except for Egypt and Morocco [42,43,44]. In Muscat, Oman, 76% of adolescent boys and 83% of girls were found to consume less than two servings of dairy/day [39], and in Lebanon, the intake of dairy was estimated to range between 0.5 and 0.7 servings/day [38]. In Iran, Shokrvash et al. (2015) reported that only 14.2% of adolescents met the recommended daily dairy serving consumption, with the average being estimated at 1.64 servings/day [45].

Few studies have assessed the intake of grains and pulses amongst adolescents. In Lebanon and Syria, grains (breads and cereals) were the highest contributor to daily EI, being estimated at 32.7% [24] and 18.4–22% EI [46], respectively. In KSA, the most frequently consumed food items amongst adolescents were grains (rice and breads), with more than 50.5% of adolescents reporting to consume rice at least once daily [47]. Studies conducted in Egypt [42], Iran [48], KSA [49], and Sudan [50], showed that 87–96% of adolescents reported daily consumption of grains (bread, rice, and other cereals). In Muscat, Oman, Waly et al. showed that the proportion of boys and girls consuming less than six servings of grains/day did not exceed one fourth of adolescents [39]. Evidence on the consumption of pulses is scarce. The proportions of adolescents reporting daily consumption of pulses were high in some countries such as Iran (53.8%) [48] and Sudan (64.9%) [51], while estimates from Bahrain [52], Palestine [53], and Jordan [54] ranged between 4% and 19%.

Evidence on the intake of meat, poultry, and fish is also scarce in the region. In Oman, the proportions of adolescents reporting to consume more than three servings of meat, poultry, and fish/day was high, ranging between 68–78% [39]. In Syria, the meat, poultry, and fish group was the second largest contributor to EI (18–21%) [46], while in Lebanon this food group provided 10.2% EI [24]. Despite the fact that the consumption of meat and poultry is frequent in this population group (Table 1), available studies suggest that the intake of fish is suboptimal. Studies conducted in KSA [47,55] reported that half of adolescents did not consume any fish or seafood during the week preceding the survey. In Syria, fish was reported to be rarely consumed by adolescents in Damascus [56], with only 6% consuming it for two times or more/week.

The consumption of high fat, high sugar, high salt foods (HFSS) is common amongst adolescents in the EMR. Table 1 shows that the proportions of adolescents reporting daily intake of SSBs ranged between 37.5% and 80%, except for Palestine and Egypt where these proportions were lower than 20%. A national study in Kuwait [57] has even reported that 43% of adolescents consumed SSBs more than once/day. Country-specific disparities were observed in the proportions of adolescents reporting daily consumption of fast foods, which ranged between 9% and 64% (Table 1). For salty and fried snacks such as potato chips and fries, the proportions of adolescents reporting daily consumption reached as high as 84% in some countries such as Iran (Table 1). Sweet snacks (Cakes/pastries and sweets/chocolates) were also found to be frequently consumed, with the proportions of adolescents reporting daily consumption ranging between 21% and 49% for sweets/chocolates, and exceeding 40% for cakes/pastries in some countries such as Sudan. In Kuwait, Honkola et al. (2006) reported that large proportions of 11–13 years adolescents consumed sweets (42%), SSBs (43%), and cakes (42.5%) several times a day, and that almost every fourth child reported consuming all of these sugary products more than once a day [58]. The frequent consumption of these HFSS suggests that these foods may have significant contributions to EI in adolescents. In Lebanon, fast foods alone were found to contribute 17% EI amongst adolescents [24], and the caloric contribution of sweets and SSBs was estimated at 10% EI and 6.5% EI, respectively [24]. In Syrian adolescents, potato chips alone provided 5% EI and the same was observed for chocolates (5% EI), while sweets and SSBs together provided close to 8% EI [46].

Studies examining water intake amongst adolescents showed that in KSA, close to 60% of 12–15 year old adolescents consumed less than 6 cups of water per day [59] and that water provided only 37% of mean daily fluid intake in 12–13 year old adolescents [60]. Similarly, in Jordan, 74% of 6–18 year olds had fewer than four cups of water daily [61]. A study conducted in Lebanon estimated mean total water intake (TWI) at 1698 mL/day amongst 9–13 years old, and showed that, compared to the adequate intake (AI) level proposed by the Institute of Medicine (IOM) [62], only 5% met the recommendations for daily TWI [63]. In the UAE, mean total daily water intake was estimated at 1116.9 mL amongst 14–18 year old adolescents compared to 922.2 mL amongst 9–13 year olds [64]. The proportion of participants who met the IOM recommendations ranged between 23% and 24% for 9–13 year olds and between 1% and 21% for 14–18 year olds [64].

### 3.2. Macronutrient Intakes Amongst School-Aged Children and Adolescents in the EMR

#### 3.2.1. School-Aged Children

Studies reporting on macronutrient intakes amongst school-aged children in the EMR are scarce. Close to 88% of 6–12 year old children in Cairo met the Recommended Dietary Allowances (RDA) for protein and [71], in Jordan, mean intake of protein ranged between 73% and 85% of the RDA amongst 5–10 year old children from Bedouin or underprivileged communities [27,72]. Another study conducted amongst five year old children from two cities in Jordan reported a high intake of total fat (34.3% EI in boys and 33.8% EI in girls) as well as a high intake of SFA (13.7–14.2% EI) [73]. Fat intake was also found to be high in Lebanese school-aged children, ranging between 35.8% and 39.7% EI, with more than half of the children exceeding the upper level for SFA intake [24,74,75]. In contrast, more than 90% of Lebanese school-aged children did not meet the recommended intakes of alpha-linolenic acid [24,74,76]. In Sudan, Alredaisy and Ibrahim (2011) showed that carbohydrates contributed 58.2% EI amongst rural school-aged children, while noting a high intake level of total fat (32.3% EI) [77]. As for countries of the GCC, a study conducted amongst 6–12 year old girls in KSA [25] showed that protein intake contributed 20.5% EI, while carbohydrates and fat provided 55% and 25.9% EI, repsectively. In the UAE, carbohydrates were reported as the main source of energy in 6–10 year old children (60% of EI) [78]. This level of carbohydrate intake was confirmed by another national study amongst children aged 6 to 13 years in the UAE, where carbohydrates provided 57.4–60.4% of daily EI [29]. It was also reported that 75–92% of participating children had fiber intakes below the requirements [74], 3.7–23% did not meet the protein requirements [74], while 28–46% exceeded the upper level for SFA [29,74].

#### 3.2.2. Adolescents

Macronutrient intakes amongst adolescents in the EMR are displayed, by country, in Table 2.

Average protein intakes reported by the various studies were within the 10–15% range suggested by the WHO. Few countries exceeded this range, and this was particularly true for Oman [79]. In many countries of the region, the intake of total fat exceeded the upper limit of 30% EI, and this was at the expense of carbohydrates.

Adequacy of macronutrient intake was investigated in some of the reviewed studies. In Iran, average protein intake was found to represent 165.4% of the RDA for protein and 154.7% of the WHO recommendations, highlighting an adequate dietary protein intake [80]. In KSA, mean nutrient adequacy ratio (NAR) of protein was estimated at 1.84, which indicates that protein intake met the dietary requirement in the majority of subjects [81]. Another study in KSA showed that mean protein intake (70.8 ± 40.6 g/day) amongst 13–18 year adolescents in Jeddah, was around 1.6 times higher than that recommended by the Academy of Nutrition and Dietetics (44 and 59 g/day for males and females respectively) [82]. In Bahrain, adolescent boys and girls consumed 1.5 times the United Kingdom’s (UK’s) Reference Nutrient intake (RNI) for protein [28,83], and in Kuwait, 86–92% of adolescents met the Acceptable Macronutrient Distribution range (AMDR) of 10–35% EI for protein [74,84]. In contrast, in Palestine, inadequate protein intake (<80% RDA) was observed amongst 15.1% of boys and 43.1% of girls aged 11–16 years [85], and in Northern Sudan, 50% of adolescents aged 13–18 years had inadequate protein intake (<80% of RDA) [86].

As for the adequacy of intake for the various subtypes of fat intake, a study conducted amongst 10–19 year old adolescents in Iran [87], showed that the average intake of SFA (10.3% EI) was close to the upper limit set by the WHO (10%) [88]. Another study in Iran reported that only a third of 6–18 year old participants, adhered to the WHO recommendations on SFA and half adhered to the recommendations related to PUFAs intakes [33]. Mirmiran et al. (2019) also reported on TFA, estimating its average intake at 2.2% EI, with only 6% of the study participants adhering to the TFA WHO recommendations of less than 1% EI [20,33]. In Lebanon, the average intake of SFA (10.7%) exceeded the WHO upper limit, while the intakes of Linoleic acid (4.8% EI) and Linolenic acid (0.13% EI) were short of the respective AMDRs of 5–10% and 0.6–1.2% EI [74,88,89]. In Palestine, the average intake of SFA amongst 11–16 year old adolescents (10.3% EI) exceeded the WHO maximal intake recommendations particularly amongst boys (12% EI) [85], while the intake of MUFAs (12.2% EI) was below the WHO recommendations of 15–20% EI [85,90]. In the UAE, Ali et al. (2013) showed that amongst 14–18 year old subjects, 12–13% of adolescents exceeded the AMDR for total fat, while 40% of girls and 60% of boys exceeded the 10% upper limit for SFA [29,74]. Similarly, in KSA, the average intake of SFA was high (11.3% EI), while that of MUFAs (10.5% EI) and PUFAs (5.8% EI) were suboptimal [82]. In Kuwait, 31–40% of adolescents exceeded the AMDR for total fat (i.e., 20–35% EI), but only 2–6% of adolescents met the AMDR for n-3 fatty acids, and 16–29% met the AMDR for n-6 fatty acids [74,84]. In Bahrain, the intakes of MUFAs and PUFAs were found to be inadequate, estimated at approximately 9–9.2% EI and 5.2–6.2% EI, respectively [28]. The PUFAs to SFA ratio of 0.6 for both girls and boys in Bahrain, was lower than the usually recommended value of 1, suggesting higher consumption of SFA compared to PUFA sources [28]. In contrast to the previous studies, the intakes of dietary fat subtypes in Tunisia were not far from recommendations, with SFA representing 9% EI, MUFAs 14% EI, and PUFAs 11% EI [91].

The majority of available studies have reported inadequate intake of dietary fiber amongst adolescents. Average dietary fiber intake was estimated at 7.5 g/day in Lebanon [89], 9 g/day in Libya [92], 11.6 g/day in Iran [41], 12.6 g/day in KSA [82], and 12.4–13.5 g/day in Bahrain, which are all considerably lower than the recommendation of the Food and Agriculture (FAO)/WHO of more than 25 g/day [90]. In countries of the GCC, average dietary fiber intake was estimated to range between 16 and 20 g/day amongst adolescents in Kuwait and between 13.6 and 20.7 g/day in the UAE [29,74,84]. The majority of adolescents (81–91% in Kuwait and 95% in UAE) did not meet the AI for fiber. Higher estimates were reported from Northern African countries in the EMR. In Morocco, fiber intake was estimated at 39.6 g/day in adolescent boys and 33.5 g/day in girls, which represented 18.8 g/1000 kcal in boys and 17.7 g/1000 kcal in girls [93,94]. These estimates are considered adequate when compared with the recommendation of 14 g fiber/1000 kcal for optimal cardiovascular health [95]. Similarly, in Tunisia, the average intake of dietary fiber intake was estimated at 36 g/day, thus exceeding the recommended level of >25 g/day [90,91].

Evidence on sugar intake amongst adolescents is very limited. A study conducted in Libya [92] reported that total sugars and FS contributed 20.4% and 12.6% of the daily EI, the latter being above the upper limit set by the WHO (10% EI) [90]. In Bahrain, the mean daily intake of total sugars was estimated to range between 98–114.6 g/day for boys and 85.5–93.8 g/day for girls, which were reported as high when compared with the maximum recommended intake of 60 g/day by the Dietary Reference Values of UK [28,83]. Adolescents aged 10–13 years from KSA were reported to consume high levels of total sugar, providing 26% EI [96]. In Iran, FS intake was estimated at close to 7% EI amongst 6–18 year old children and adolescents, with 81% of boys and 84% of girls adhering to the FS WHO recommendations [20,33].

### 3.3. Micronutrient Intakes Amongst School-Aged Children and Adolescents in the EMR

#### 3.3.1. School-Aged Children

In Jordan, a study conducted amongst 5–6 year old children [73] showed that mean intakes of several vitamins were below their respective Dietary Reference Intakes (DRIs). More specifically, vitamins A and B12 represented 60–70% of the respective DRIs and similar values were observed for folate (73–75% DRI) and vitamin C (57–60% DRI). Inadequate intakes of calcium (64–68% DRI), iron (66–73% DRI), and zinc (56–60% DRI) were also reported [73]. Other studies conducted in Jordan, especially amongst Bedouins and children from underprivileged areas, reported that mean intakes of iron, calcium, and vitamin A represented 50%, 70%, and 65–80% of their respective RDAs [27,72,102,103]. Similarly, in school-aged children in Lebanon, 84–95%, 73–88%, and 35% did not meet two-thirds of the RDA for vitamin D, calcium, and iron, respectively [76,102,104]. In KSA, mean calcium intakes in children aged 7–12 years old did not exceed 60% of the RDA, and mean vitamin D intake represented only 23% of RDA [102,105]. In the UAE, a national study showed that more than 76% of 6–8 year old children did not meet the respective Estimated Average Requirement (EAR) level for vitamin A, while for vitamin D and vitamin E, more than 93% of 6–8 year old children did not meet the EAR value [29]. In addition, 26% of boys and 43% of girls did not meet the EAR intake level for folate [29]. In Lebanon, a study conducted amongst 5–12 year olds showed that 23% and 95% did not meet 2/3rd of the RDA for vitamin E and D, respectively [76]. In Egypt, 44–76% of 6–12 years old children did not meet 50% RDA for vitamin A [71], and close to a third did not meet 50% RDA for iron and calcium.

High intakes of sodium (Na) coupled with low intakes of potassium (K) were reported by studies in the region. In Iran, mean intakes of Na and K amongst 3–10 year old children were 2017 mg/day and 1119 mg/day, respectively [26], while the recommended intakes in this age group ranged from <1500 to <1900 mg/day and 3000–3800 mg/day, respectively [9]. In Kuwait, 64% of girls and 71% of boys aged 4–8 years, exceeded the Tolerable Upper Intake Level for Na (1900 mg/day) of the IOM [84]. In Morocco, average intake of Na was estimated at 1800 mg/day amongst 6–8 year old children, with 46.7% exceeding the IOM upper limit [106].

#### 3.3.2. Adolescents

**Figure 3:**
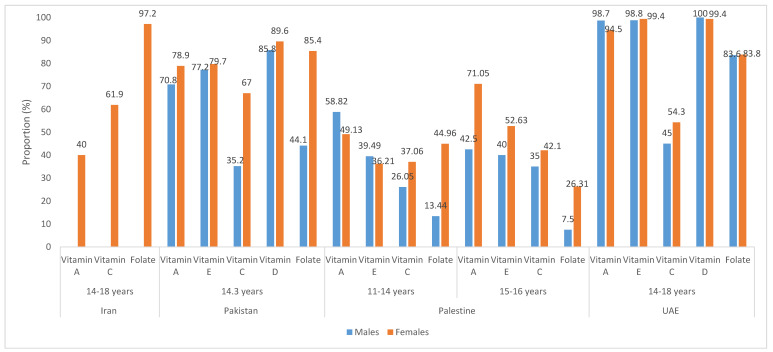
Proportion of adolescents not meeting the recommendations for vitamins A, E, C, D and folate. The criteria used to assess the proportion of adolescents not meeting the recommendation, are as follows: Iran, Recommended Dietary Allowances (RDAs); Pakistan, Estimated Average Requirement (EAR)/Average Intake (AI); Palestine, <80% RDA; United Arab Emirates (UAE), <EAR.

Figure 3 illustrates the proportions of adolescents not meeting the recommended intake levels of vitamins A, C, E, D and folate [29,48,85,99]. It is important to note that the data reported by the various studies is not readily comparable given that different studies have used different benchmarks to define nutrient adequacy. Taken together, the data underline suboptimal intakes for the micronutrients in Iran, Pakistan, Palestine and the UAE, with high proportions of adolescents not meeting the recommended nutrient intake levels. Similarly, in KSA, 63% and 87% of adolescents (9–18 year olds) had intakes below EAR for vitamin A and E, respectively [107]. In Lebanon 55.3% of Lebanese children and adolescents (6–19 year olds) did not meet 2/3rd the RDA for vitamin A, with 23–26% also not meeting 2/3rd the RDA for vitamins C and E [89]. Inadequate intakes for thiamin, riboflavin, pyridoxine, vitamin B12 were also reported by some studies in the region [29,48,85].

**Figure 4:**
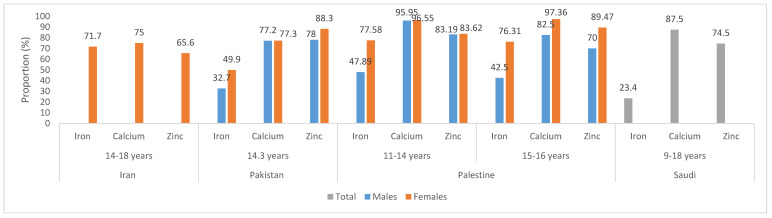
Proportion of adolescents not meeting the recommendation for iron, calcium and zinc. The criteria used to assess the proportion of adolescents not meeting the recommendations, are as follows: Iran, Recommended Dietary Allowances (RDAs); Pakistan, Estimated Average Requirement (EAR)/Average Intake (AI); Palestine, <80% RDA; United Arab Emirates (UAE), <EAR.

Figure 4 displays the proportions of adolescents not meeting the recommended intake levels of iron, calcium and zinc. The data suggest that high proportions of adolescents do not meet the recommendations for these nutrients in Iran, Pakistan, Palestine and KSA [48,85,99,107]. A study conducted in Lebanon of 6–19 year old children and adolescents showed that 27% and 36% do not meet 2/3rd the RDA for zinc and iron, respectively, while 77% do not meet two-thirds of the RDA for calcium [89].

Available evidence suggests that sodium (Na) intakes are high in this age group. In Kuwait, 46–61% of females and 73–80% of males aged 9–18 years exceeded the Tolerable Upper Intake Level for Na (2200–2300 mg/day) set by the IOM [84]. Mean Na intakes amongst adolescents in KSA ranged between 2209 and 2250 mg/day, exceeding the AI level (1700 mg/day) and the WHO Upper limit (2000 mg/day) [82,107]. In Morocco, Na intake was estimated at 2193.4 mg/day and 2138.0 mg/day in those aged 9–13 years and 14–18 years, respectively with 26.7–49.3% exceeding the upper intake level set by the IOM [106]. In parallel, low potassium intakes (K) were reported. In Pakistan, 45% of boys and 51% of girls had intakes below the EAR for potassium [99]. In KSA, mean intakes of K ranged between 1530 mg and 1961 mg/day, thus being inferior to the AI of 4500–4700 mg/day [82,107], and 87% of adolescents had intakes less than the AI level for K [107]. In Tunisia, mean K intake was estimated at 1044.5–1053.8 mg/day amongst 15–19 year adolescents [91], and in Morocco, 75% of children and adolescents aged 6–18 year old children and adolescents [106] consumed less than the AI of K [106]. Low intakes of phosphorous, magnesium, manganese, copper and selenium have also been reported by some studies in the region [76,85,99,107].

## 4. Discussion

The majority of available studies amongst children and adolescents in the EMR have documented a low intake of fruits, vegetables and fiber, inadequate consumption of water, milk and dairy products, coupled with a high intake of SSBs, and a frequent consumption of energy-dense, nutrient poor foods such as sweet and savory snacks. High intakes of fat and SFA were also observed in several studies conducted in the region, coupled with a number of micronutrient inadequacies, particularly low intakes of calcium, iron, and zinc and vitamins A, D, C and folate.

These food consumption and dietary intake patterns may be linked with suboptimal nutritional status and increased risk for obesity and cardiometabolic risk factors. This is of concern to the EMR given that the region harbors a “triple” burden of malnutrition in children and adolescents, characterized by the persistence of undernutrition, an alarming escalating burden of overweight and obesity, and a high prevalence of micronutrient deficiencies [108,109]. A recent review showed that the estimated weighted regional averages for stunting, wasting and underweight were 28%, 8.69% and 18%, respectively [109]. The prevalence of anemia was found to range between 16% and 81% amongst school-aged children and adolescents in countries of the region, while that of vitamin D deficiency ranged between 21% and 83% [109]. Several countries in the EMR reported an increasing trend in the prevalence of overweight and obesity amongst school-aged children and adolescents, the highest increases being reported from Iran [110,111], Lebanon [112], Qatar [113,114], Saudi Arabia [115,116,117], Tunisia [118] and Bahrain [119,120]. The prevalence of obesity amongst school-aged children and adolescents reached as high as 29.6% in Kuwait [121] and 21.7% in Bahrain [122]. The escalating and high prevalence of child and adolescent obesity raises questions about its implications for disease burden in the region, given its association with metabolic syndrome, insulin resistance, hypertension, dyslipidemia and hyperglycemia [121,123]. Studies conducted in various EMR countries reported a high prevalence of metabolic syndrome in obese children and adolescents, ranging between 15% and 30% in countries of the Levant and reaching as high as 44% in countries of the GCC, such as the UAE [7,8,124,125,126]. With those younger than 14 years representing approximately 30% of the population of the EMR, these estimates do not bode well for the future health and well-being of the population, and the development and building of productive societies [108].

The faulty dietary practices documented in this review, and which are in many instances similar to those reported by Keats et al. in low and middle income countries [19], may at least partially explain the increase in pediatric and adolescent adiposity and the persistence of undernutrition in the EMR. For instance, low intakes of fruit and vegetables may be a risk factor for obesity. In fact, available evidence suggests that adequate consumption of fruits and vegetables is usually associated with lower EI and higher intakes of dietary fiber which, through colonic, intrinsic, and/or hormonal effects may be associated with increased satiety, increased fat oxidation, and increased insulin sensitivity, all of which may contribute to the prevention of obesity and metabolic abnormalities [95,127,128,129]. In addition, high intake of SSBs can promote weight gain through their low satiety, incomplete compensatory reduction in EI at subsequent meals and high content of added sugar [130]. On average, SSBs provide approximately 140–150 calories and 35.0–37.5 g of sugar per 12-oz serving [131]. In addition, fructose from sucrose or from high corn fructose syrup has been linked with the development of visceral adiposity and ectopic fat deposition [132,133,134,135]. Several societies and organizations including the American Academy of Pediatrics and the WHO have advocated for reductions in the intake of SSBs to help prevent obesity and enhance overall health [136]. The observed high intakes of fat and SFA may also be linked with the burden of obesity in the region, given their high energy density and the promotion of adipogenesis [137]. The overall food consumption pattern that is low in fruit and vegetables, while being high in high fat, high sugar foods and beverages, is a hallmark of the western dietary pattern, which has been repetitively shown to be associated with increased adiposity risk [138]. At the same time, such food consumption and dietary patterns are associated with low dietary diversity, insufficient consumption of nutrient dense foods and suboptimal micronutrient intakes, which may at least partially explain the persistent burden of undernutrition and micronutrient deficiencies in countries of the EMR. The observed high intake of sodium, coupled with low intakes of potassium, is recognized as a risk factor for raised blood pressure in childhood and adolescents, and may increase the risk for hypertension and cardiovascular disease later in life [139].

Numerous factors may influence the diets of children and adolescents. These comprise both individual and socio-cultural factors as well as economic and environmental factors [140]. At the individual level, poor nutritional knowledge may be associated with unhealthy dietary practices. Studies conducted in countries of the region have documented significant nutrition knowledge gaps in children and adolescents, especially in what relates to nutrient sources, the identification of healthy snacks and diet-disease relationships [38,141,142]. Other factors such as personal likings, taste preferences, self-efficacy, and body image [143,144,145,146] may also play an important role in shaping the dietary practices in this age group [140]. Children and adolescents are also highly affected by the food environment, including the affordability, availability, and access to foods [140,147,148,149,150,151]. Marketing and advertising of ultra-processed foods with a high content of fat, sugar and/or salt to children and adolescents was also recognized as a factor that promotes suboptimal diets amongst children and adolescents [140,152]. Studies conducted in countries of the region have shown that the marketing of ultra-processed, nutrient-depleted foods is highly common on television, during children’s programs and/or children’s viewing time [153,154].

The WHO developed several standards and guidelines for health policies, strategies and interventions aimed at improving the nutrition status of children and adolescents. Aligned with the SDGs, and guided by the Global Strategy for Women’s Children’s and Adolescent’s Health (2016–2030), the WHO Child and Adolescent Health and Nutrition program aims to translate global nutrition guidelines into actions to address the double burden of malnutrition in various countries of the world, build capacity for the monitoring of health and nutrition status, and develop evidence-based policies that contribute to the improvement of health and nutrition of children and adolescents [2]. The WHO has also established the nutrition-friendly school initiative that provides a framework for ensuring integrated school-based programs that address the double-burden of nutrition-related ill health [155], and has further articulated priorities related to adolescent nutrition in the Global Accelerated Action for the Health of Adolescents [156]. In line with its mandate, the regional office of the WHO has been active in shaping the policy environment in Member States, emphasizing the need for policies and initiatives that promote a healthier food environment for the population, with a focus on children and adolescents. The WHO EMR published, in 2018, a set of recommendations on the marketing of food and non-alcoholic beverages to children in the region [157]. The recommendations aim to guide Member States on the promotion of responsible marketing and the regulation of the marketing of foods and beverages that are high in saturated fat, trans fat, free sugar or salt to children [157]. Countries that have adopted legislation that contributes to the implementation of these recommendations include Egypt, Iran and Saudi Arabia. The WHO Regional Strategy (2020–2030) [158] also provides a comprehensive framework for regional and national efforts to reach the various targets on nutrition, including the promotion of healthier diets amongst children and adolescents. It has mapped existent nutrition policies in the region and showed that the main action areas included in nutrition policies across the region are infant and young child nutrition (84%), school health and nutrition programs (84%), healthy diet awareness (84%), vitamin and mineral nutrition (79%), acute malnutrition (53%) and nutrition and infectious diseases (37%). In addition, the Regional framework for action on obesity prevention 2019–2023 [159] provides a set of strategic interventions and progress indicators related to fiscal measures, public procurement, food supply and trade, food labeling, physical activity promotion, mass media campaigns, and product reformulation, coupled with continuous assessment and monitoring to help Member states in their obesity prevention efforts. Moreover, the WHO EMR office has issued several policy statements related to lowering sugar, fat and salt intakes in countries of the EMR [160,161,162], providing a road map for countries of the region for the progressive and sustainable reduction in national intakes of sugar, fat and salt. Despite the active policy response in several countries of the EMR, recent reports [140,163] are highlighting the need for a broader food system approach in order to improve the diets of children and adolescents. To better address the nutritional needs of children and adolescents, the food system should be leveraged and aligned along its four determinants (food supply chains, external food environments, personal food environments, and behaviors of caregivers, children and adolescents) to improve the quality of the diet in this age group [140].

## 5. Missing Knowledge and Future Research

Although this review has provided valuable insight into food consumption patterns and dietary intakes amongst school-aged children and adolescents in the EMR, it has identified several challenges and gaps in the existing dietary assessment studies. In particular, findings on food consumption patterns were often limited by the scarcity of data, particularly in school-aged children. This knowledge gap has been reported by the United Nations Children’s Fund (UNICEF) [140], stating that the age group of school-aged children is often missing from health and nutrition surveys. The fact that most of the available studies have reported on daily or weekly frequency of intake, rather than quantifying consumption, has also often limited the interpretability of the data. Another challenge stemmed from the fact that most of the available studies were conducted at a small or regional scale, with only few countries having conducted national surveys on food consumption in children and adolescents. Political instabilities and turmoil, coupled with limited research funding, are amongst the challenges that some countries of the region are facing and that may contribute to the scarcity of nationally representative data. It is also important to mention that dietary assessment in many countries of the region may be limited by the availability of complete and up-to-date food composition tables, particularly for traditional foods and composite dishes, highlighting the crucial need for concerted efforts in this domain. The findings of this review may also be limited by the fact that different dietary assessment methods (food frequency questionnaires, FFQ; 24-hr recalls, 24-HR; dietary records) were used by the various countries/studies, which may impact the comparability of the generated food consumption data [108].

Based on the work undertaken in this paper, opportunities for future research include regional collaborations to: (1) consolidate and update food composition tables, with a focus on culture-specific foods and composite dishes; (2) conduct nationwide dietary surveys on children and adolescents using validated and standardized approaches and methodologies; (3) contribute to a better characterization of food consumption patterns and dietary intakes in under-represented age groups, such as school-aged children; (4) assess the intake and sources of free sugar; (5) assess the intake and sources of trans fat; and (6) gain a better understanding of factors that may be associated with unhealthy food consumption patterns in the EMR. These priorities may guide policy makers, researchers, funding agencies, and non-governmental organizations in tackling the identified knowledge gaps and developing culture specific and evidence-based intervention strategies aimed at improving the nutritional status of children in the EMR.

## 6. Conclusions

This review contributes toward the characterization of food consumption patterns and dietary intakes amongst children and adolescents in the EMR. The findings highlighted poor dietary habits in these age groups characterized by low intakes of fruit, vegetables, and dairy coupled with high intakes of SSBs and frequent consumption of sweets and savory snacks. High intakes of fat and saturated fat were observed, while the intakes of several micronutrients were inadequate. These suboptimal food consumption and dietary intake patterns represent a public health concern, given that the triple burden of malnutrition continues to plague most countries of the region. The findings of this review have therefore broad implications for developing public health strategies and policies to improve the diet of children and adolescents in the EMR. Acknowledging the impact that nutrition may have on building societies and transforming the lives of children, adolescents and their families, there is a crucial need for a food system approach in developing and implementing national and regional policies and interventions aimed at improving the diet of children and adolescents. Such interventions will not only enhance the diet and nutritional status of young people, but will also pave the way towards the achievement of many sustainable development goal targets, including ensuring healthy lives, promoting life-long learning, improving economic growth and building inclusive societies [164].

## Figures and Tables

**Table 1:** Proportions of adolescents reporting daily consumption (unless otherwise indicated) of various food groups * in countries of the Eastern Mediterranean Region.

Country	Fruits	Vegetables	Dairy	Meat, Poultry, and Fish	Fast Foods	SSBs	Sweet Snacks (Including Confectionary)		Salty and Fried Snacks
							Cakes/Pastries	Sweets/Chocolates	
Bahrain [52] (National)	25.3%	26.3%	37.1%	Meat: 20% Fish: 6.9% Poultry: 18.2%	14.4%	42.2%	--	Sweets: 31.4% Chocolates: 32%	--
Egypt [42]	29%	78.2%	58.9%	--	64.6%	19.7%	--	--	--
Iran [48]	16.2%		16.3%	Meat: 31.9%	--	75.4%	45.5%		84.7%
Iraq [36]	M: 24.3% F: 46.3%	M: 46.1% F: 62.3%	Milk: M: 37.2% F: 35.2%	--	>3 days/w: M: 37.1% F: 24.9%	>3 days/w: M: 66.9% F: 60.4%	>3 days/w: M: 46.1% F: 55%	>3 days/w: M: 43.5% F: 52.2%	>3 days/w: M: 51% F: 64.6%
Jordan [54]	20%	43%	--	1–3 times/w: Meat: 47% Fish: 54% 4–6 times/w: Poultry: 40%	1–3 times/w: 57%	--	--	Chocolates: 40%	--
Kuwait [57]	M: 17.5% F: 11.8%	M: 26% F: 22.1%	M: 36.3% F: 25.3%	--	M: 9.4% F: 10.4%	M: 42.2% F: 37.5%	M: 7% F: 14.7%	M: 21.1% F: 35.6%	Potato fries/chips: M: 9.4%; F: 12.4%
Kuwait [58] (National)	--	--	--	--	--	>1 time/d: 43%	>1 time/d: 42.5%	>1 time/d: 42%	--
Morocco [43,44]	M: 18.7% F: 20.4%	M: 33.1% F: 42.5%	M: 78.1% F: 76.9%	--	>3 times/w: M: 15% F: 12.9%	>3 times/w: M: 37.5% F: 41.9%	>3 times/w: M: 66.9% F: 79%	3 times/w: M: 66.9% F: 72%	>3 times/w: Potato fries/chips: M: 18.8%; F: 29%
Palestine [53]	M: 11.6% F: 16.2%	M: 27.6% F: 34.1%	Milk: M: 33.7% F: 29.9% Yogurt: M: 19.8% F: 20.8%	>3 times/w: Meat: M: 7.3% F: 7.9% Poultry: M: 3.4% F: 3.8%	--	M: 6.2% F: 9.3%	Cookies: M: 14.6% F: 19.6%	Chocolates: M: 10.7% F: 17%	--
Palestine [65] (National)	31%	45%	Milk: 22%	Meat and poultry: 16%	--	24%	35%		--
Palestine [37]	M: 58.9% F: 55.2%	M: 72.8% F:73.8%	Milk: M: 32.9% F: 18.3% Yogurt: M: 31.8% F: 28.3%	Meat: M: 11.4% F: 10.7% Poultry: M: 11.9% F: 12.2%	--	M: 39.6% F: 28.4%	M: 42.3% F: 49.2%		Salty snacks: M: 50.3% F: 61.5% Fried potatoes: M: 20.5% F: 23.8%
Qatar [66]	13.9%	20.3%	24.1%	--	≥4 days/w: 27.3%	≥4 days/w: 48.8%	≥4 days/w: 24.5%	≥4 days/w: 49.4%	≥4 days/w: 28.7%
Saudi [67]	M: 16% F: 9.6%	M: 23.3% F: 22.3%	Milk: M: 33.2% F: 25.1%	--	>3 days/w: M: 30.2% F: 24.9%	>3 days/w: M: 67.3% F: 57.4%	>3 days/w: M: 24.8% F: 28.8%	>3 days/w: M: 37.3% F: 52.6%	>3 days/w: M: 25% F: 30.7%
Sudan [50]	M: 33.5% F: 31.9%	M: 70.1% F: 69.7%	--	Meat: M: 55.7% F: 60.7%	--	M: 43.9% F: 44.8%	Sweets: M: 55.4% F: 60.6%	--	Crisps: M: 38.7% F: 39.2% Other salty snacks: M: 42.9% F: 32%
Sudan [51]	>4 times/w: 30.1%	>4 times/w: 63.9%	>4 times/w: 58.1%	>4 times/w: Meat: 59.1% Fish: 11.4% Poultry: 27.8%	>4 times/w: 26.6%	>4 times/w: 43.4%	>4 times/w: 37.1%	>4 times/w: Chocolates: 30.1%	--
Sudan [68]	--	--	--	--	--	80.6%	Dessert: 69.3% Sweet biscuits: 65.3%	Chocolates: 80% Popsicles: 61.4% Sweets: 48.7%	--
UAE [40]	--	--	--	--	≥1 time/w: M: 77.3% F: 81.4%	≥1 time/d: M: 41.1% F: 34%	--	--	--

**Abbreviations:** SSBs: sugar-sweetened beverages; M: males; F: females; w: week; d: day; UAE: United Arab Emirates. Salty snacks may include potato chips, French fries, popcorn, crackers. *: Food items categorization was adopted and modified based on a review by Keats et al., 2018 [19,69,70]. The following categories were not included in the table due to limited data: Grains, white roots, tubers and plantains; Pulses (beans, peas, lentils); Nuts and seeds; Eggs; Oils and fats.

**Table 2:** Macronutrients intakes amongst adolescents in countries of the Eastern Mediterranean region.

Country	Study Area	Study Population	Dietary Assessment	CHO (%EI)	Protein (%EI)	Fat (%EI)
Bahrain [28]	11 regions of Bahrain	11–18 year old children and adolescents; *n* = 496	24-HR	M: 45–52.5; F: 52–53	M: 15.5–15.9; F: 15–15.4	M: 31.1–32.5; F: 32.7–33.9
Egypt [97]	Sohag	12–18 year old adolescents; *n* = 300	24-HR	59.1–61.3	15.7–15.9	26.9–28.5
Iran [80]	Lahijan, Northern Iran	14–17 year old girls; *n* = 400	24-HR	59.3	11.9	28.8
Iran [87]	Tehan	10–19 year old girls; *n* = 717	FFQ	--	--	30.6
Iran [48]	Sistan and Baluchistan	14–18 year old girls; *n* = 753	2-day 24-HR	54	14	31.9
Iran [33]	Tehan	6–18 year old; *n* = 424	FFQ	M: 57.2; F: 56.8	M: 12.9; F: 13.2	M: 32.1; F: 32.4
Iran [34]	Isfahan	Primary school and junior high school pupils; *n* = 4700	FFQ	64.1	12	23.8
Kuwait [98]	Different regions in Kuwait	8, 13 and 17 year old students; *n* = 588	Questionnaire	60.6	13	32.2
Kuwait [84]	National	9–18 year old children and adolescents; *n* = 614	24-HR	M: 53–54; F: 53	M: 15; F: 14–15	M: 31–32; F: 32–33
Lebanon [24]	National	12–19 year old adolescents; *n* = 498	24-HR	51.1	13.5	36.2
Lebanon [89]	National	12–19 year old children and adolescents; *n* = 3394	24-HR	51.4	13.4	36
Libya [92]	Benghazi	12 year old adolescents; *n* = 180	3-day food record	54	15.7	30.2
Morocco [93,94]	Ouarzazate	15–18 year old adolescents; *n* = 327	3-day food record	M: 58.8; F: 56.6	M: 12.9; F: 12.1	M: 28.3; F: 31.3
Oman [79]	Muscat	15–18 year old adolescents; *n* = 802	FFQ	M: 55.5; F: 51.1	M: 22.5; F: 18.7	M: 30.4; F: 22.6
Pakistan [99]	Sialkot	Mean age: 14.3 years; *n* = 328	3-day food record	51.5	12.5	36.3
Pakistan [100]	National	6–16 year old children and adolescents; *n* = 237	24-HR	60–74	10–12	18–32
Palestine [85]	East Jerusalem	11–16 year old adolescents; *n* = 313	24-HR	54	12.7	34.7
Saudi-Arabia [101]	Riyadh and Dawadami	17–19 year old adolescents; *n* = 600	24-HR	R: 54.4; U: 54.3	R: 16.9; U: 15.1	R: 29.5; U: 32.8
Saudi-Arabia [82]	Jeddah	13–18 year old adolescents; *n* = 239	3-day 24-HR	56.6	13	30.5
Sudan [86]	Northern State	10–19 year old adolescents; *n* = 401	24-HR	77.4	12.6	9.9
Tunisia [91]	3 regions in Tunisia	15–19 year old adolescents; *n* = 1019	FFQ	52	12	36
UAE [78]	National	11–18 year old adolescents; *n* = 276	24-HR	M: 59.1; F: 58.2	M: 16; F: 14.9	M: 25.8; F: 27.8
UAE [29]	National	11–18 year old adolescents; *n* = 276	24-HR	--	M: 15–16; F: 14.7–15.3	M: 25.2–26.7; F: 27.6–27.9

**Abbreviations:** CHO: carbohydrates; EI: energy intake; M: males; F: females; R: rural; U: urban; 24-HR: 24-hr dietary recall; FFQ: food frequency questionnaire.

## References

[1] World Health Organization Regional Office for Europe (2017). Fact Sheets on Sustainable Development Goals: Health Targets: Child and Adolescent Health.

[2] World Health Organization Regional Office for Africa Child and Adolescent Health and Nutrition (CAN) Program. https://www.afro.who.int/about-us/programmes-clusters/CAN.

[3] Institute of Medicine (2007). Nutrition-Related Health Concerns, Dietary Intakes, and Eating Behaviors of Children and Adolescents. Nutrition Standards for Foods in Schools: Leading the Way Toward Healthier Youth.

[4] Story M., Holt K., Sofka D. (2002). Bright Futures in Practice.

[5] Daniels S.R. (2006). The consequences of childhood overweight and obesity. Future Child.

[6] Institute of Medicine (2005). Preventing Childhood Obesity: Health in the Balance.

[7] Nasreddine L., Naja F., Tabet M., Habbal M.-Z., El-Aily A., Haikal C., Sidani S., Adra N., Hwalla N. (2012). Obesity is associated with insulin resistance and components of the metabolic syndrome in Lebanese adolescents. Ann. Hum. Biol..

[8] Nasreddine L., Ouaijan K., Mansour M., Adra N., Sinno D., Hwalla N. (2010). Metabolic syndrome and insulin resistance in obese prepubertal children in Lebanon: A primary health concern. Ann. Nutr. Metab..

[9] Gidding S.S., Dennison B.A., Birch L.L., Daniels S.R., Gilman M.W., Lichtenstein A.H., Rattay K.T., Steinberger J., Stettler N., Van Horn L. (2005). Dietary recommendations for children and adolescents: A guide for practitioners: Consensus statement from the American Heart Association. Circulation.

[10] World Health Organization (2010). Technical paper. Regional strategy on nutrition 2010–2019. Regional Committee for the Eastern Mediterranean. Fifty-Seventh Session. Agenda Item 4 (b).

[11] Nasreddine L., Naja F., Sibai A.-M., Helou K., Adra N., Hwalla N. (2014). Trends in nutritional intakes and nutrition-related cardiovascular disease risk factors in Lebanon: The need for immediate action. Leb. Med. J..

[12] Popkin B.M., Adair L.S., Ng S.W. (2012). Global nutrition transition and the pandemic of obesity in developing countries. Nutr. Rev..

[13] World Health Organization Regional Office for the Eastern Mediterranean Nutrition. http://www.emro.who.int/health-topics/nutrition/index.html.

[14] Rahim H.F.A., Sibai A., Khader Y., Hwalla N., Fadhil I., Alsiyabi H., Mataria A., Mendis S., Mokdad A.H., Husseini A. (2014). Non-communicable diseases in the Arab world. Lancet.

[15] Moghames P., Hammami N., Hwalla N., Yazbeck N., Shoaib H., Nasreddine L., Naja F. (2015). Validity and reliability of a food frequency questionnaire to estimate dietary intake among Lebanese children. Nutr. J..

[16] Bagchi K. (2008). Nutrition in the eastern Mediterranean region of the World Health Organization. East. Mediterr. Health J..

[17] World Health Organization Regional Office for the Eastern Mediterranean Countries in the Eastern Mediterranean Region. http://www.emro.who.int/countries.html.

[18] World Health Organization Global School-Based Student Health Survey (GSHS). https://www.who.int/ncds/surveillance/gshs/factsheets/en/.

[19] Keats E.C., Rappaport A.I., Shah S., Oh C., Jain R., Bhutta Z.A. (2018). The dietary intake and practices of adolescent girls in low-and middle-income countries: A systematic review. Nutrients.

[20] World Health Organization (2015). Healthy Diet FACT SHEET N°394.

[21] Abbass M.M., Mahmoud S.A., El Moshy S., Rady D., AbuBakr N., Radwan I.A., Ahmed A., Abdou A., Al Jawaldeh A. (2019). The prevalence of dental caries among Egyptian children and adolescences and its association with age, socioeconomic status, dietary habits and other risk factors. A cross-sectional study. F1000Research.

[22] Hashim R., Williams S.M., Murray Thomson W. (2009). Diet and caries experience among preschool children in Ajman, United Arab Emirates. Eur. J. Oral. Sci..

[23] Jaghasi I., Hatahet W., Dashash M. (2012). Dietary patterns and oral health in schoolchildren from Damascus, Syrian Arab Republic. East. Mediterr. Health J..

[24] Nasreddine L., Naja F., Akl C., Chamieh M.C., Karam S., Sibai A.-M., Hwalla N. (2014). Dietary, lifestyle, and socio-economic correlates of overweight, obesity and central obesity in Lebanese children and adolescents. Nutrients.

[25] Hasanein M.A., Jawad S.H.A. (2014). Prevalence of obesity and risk factors among female school-aged children in primary school in Madinah Munawarah. Life Sci. J..

[26] Kelishadi R., Gheisari A., Zare N., Farajian S., Shariatinejad K. (2013). Salt intake and the association with blood pressure in young Iranian children: First report from the middle East and north Africa. Int. J. Prev. Med..

[27] Khatib I., Elmadfa I. (2009). High prevalence rates of anemia, vitamin A deficiency and stunting imperil the health status of Bedouin schoolchildren in North Badia, Jordan. Ann. Nutr. Metab..

[28] Gharib N., Rasheed P. (2011). Energy and macronutrient intake and dietary pattern among school children in Bahrain: A cross-sectional study. Nutr. J..

[29] Ali H.I., Ng S.W., Zaghloul S., Harrison G.G., Qazaq H.S., El Sadig M., Yeatts K. (2013). High proportion of 6 to 18-year-old children and adolescents in the United Arab Emirates are not meeting dietary recommendations. Nutr. Res..

[30] Alsubaie A.S.R. (2018). Intake of fruit, vegetables and milk products and correlates among school boys in Saudi Arabia. Int. J. Adolesc. Med. Health.

[31] Hassan A.S., Al-Dosari S.N. (2008). Breakfast habits and snacks consumed at school among Qatari schoolchildren aged 9–10 years. Nutr. Food Sci..

[32] Amini M., Dadkhah-Piraghaj M., Abtahi M., Abdollahi M., Houshiarrad A., Kimiagar M. (2014). Nutritional assessment for primary school children in Tehran: An evaluation of dietary pattern with emphasis on snacks and meals consumption. Int. J. Prev. Med..

[33] Mirmiran P., Ziadlou M., Karimi S., Hosseini-Esfahani F., Azizi F. (2019). The association of dietary patterns and adherence to WHO healthy diet with metabolic syndrome in children and adolescents: Tehran lipid and glucose study. BMC Public Health.

[34] Naeeni M.M., Jafari S., Fouladgar M., Heidari K., Farajzadegan Z., Fakhri M., Karami P., Omidi R. (2014). Nutritional knowledge, practice, and dietary habits among school children and adolescents. Int. J. Prev. Med..

[35] Kelishadi R., Ardalan G., Gheiratmand R., Gouya M.M., Razaghi E.M., Delavari A., Majdzadeh R., Heshmat R., Motaghian M., Barekati H. (2007). Association of physical activity and dietary behaviours in relation to the body mass index in a national sample of Iranian children and adolescents: CASPIAN Study. Bull. World Health Organ..

[36] Musaiger A.O., Al-Mufty B.A., Al-Hazzaa H.M. (2014). Eating habits, inactivity, and sedentary behavior among adolescents in Iraq: Sex differences in the hidden risks of noncommunicable diseases. Food Nutr. Bull..

[37] Mikki N., Abdul-Rahim H.F., Shi Z., Holmboe-Ottesen G. (2010). Dietary habits of Palestinian adolescents and associated sociodemographic characteristics in Ramallah, Nablus and Hebron governorates. Public Health Nutr..

[38] Nabhani-Zeidan M., Naja F., Nasreddine L. (2011). Dietary intake and nutrition-related knowledge in a sample of Lebanese adolescents of contrasting socioeconomic status. Food Nutr. Bull..

[39] Waly I., Zayed K., Al Haddabi B. (2017). Obesity, eating habits and sedentary behaviour of Omani young adolescents: A cross-sectional study. EC Nutr..

[40] Makansi N., Allison P., Awad M., Bedos C. (2018). Fruit and vegetable intake among Emirati adolescents: A mixed methods study. East. Mediterr. Health J..

[41] Hejazi N., Mazloom Z. (2009). Socioeconomic status, youth’s eating patterns and meals consumed away from home. Pak. J. Biol. Sci..

[42] Abdel-Hady D., El-Gilany A.-H., Sarraf B. (2014). Dietary habits of adolescent students in Mansoura, Egypt. Int. J. Collab. Res. Intern. Med. Public Health.

[43] El Achhab Y., Marfa A., Echarbaoui I., Chater R., El-Haidani A., Filali-Zegzouti Y. (2018). Physical inactivity, sedentary behaviors and dietary habits among Moroccan adolescents in secondary school. Sci. Sport.

[44] Hamrani A., Mehdad S., El Kari K., El Hamdouchi A., El Menchawy I., Belghiti H., El Mzibri M., Musaiger A.O., Al-Hazzaa H.M., Hills A.P. (2015). Physical activity and dietary habits among Moroccan adolescents. Public Health Nutr..

[45] Shokrvash B., Salehi L., Akbari M.H., Mamagani M.E., Nedjat S., Asghari M., Majlessi F., Montazeri A. (2015). Social support and dairy products intake among adolescents: A study from Iran. BMC Public Health.

[46] Nasreddine L., Mehio-Sibai A., Mrayati M., Adra N., Hwalla N. (2010). Adolescent obesity in Syria: Prevalence and associated factors. Child Care Health Dev..

[47] Mahfouz A.A., Shatoor A.S., Hassanein M.A., Mohamed A., Farheen A. (2012). Gender differences in cardiovascular risk factors among adolescents in Aseer Region, southwestern Saudi Arabia. J. Saudi Heart Assoc..

[48] Montazerifar F., Karajibani M., Dashipour A.R. (2012). Evaluation of dietary intake and food patterns of adolescent girls in Sistan and Baluchistan Province, Iran. Funct. Foods Health Dis..

[49] Sachithananthan V., Gad N. (2016). A Study on the Frequency of Food Consumption and Its Relationship to BMI in School Children and Adolescents in Abha City, KSA. Cur. Res. Nutr. Food Sci..

[50] Moukhyer M.E., van Eijk J.T., De Vries N.K., Bosma H. (2008). Health-related behaviors of Sudanese adolescents. Educ. Health.

[51] Musaiger A.O., Nabag F.O., Al-Mannai M. (2016). Obesity, dietary habits, and sedentary behaviors among adolescents in Sudan: Alarming risk factors for chronic diseases in a poor country. Food Nutr. Bull..

[52] Musaiger A., Bader Z., Al-Roomi K., D’Souza R. (2011). Dietary and lifestyle habits amongst adolescents in Bahrain. Food Nutr. Res..

[53] Abudayya A.H., Stigum H., Shi Z., Abed Y., Holmboe-Ottesen G. (2009). Sociodemographic correlates of food habits among school adolescents (12–15 year) in North Gaza Strip. BMC Public Health.

[54] Dalky H.F., Al Momani M.H., Al-Drabaah T.K., Jarrah S. (2017). Eating habits and associated factors among adolescent students in Jordan. Clin. Nurs. Res..

[55] Mahfouz A.A., Abdelmoneim I., Khan M.Y., Daffalla A.A., Diab M.M., Al-Gelban K.S., Moussa H. (2008). Obesity and related behaviors among adolescent school boys in Abha City, Southwestern Saudi Arabia. J. Trop. Pediatr..

[56] Musaiger A.O., Kalam F. (2014). Dietary habits and lifestyle among adolescents in Damascus, Syria. Ann. Agric. Environ. Med..

[57] Allafi A., Al-Haifi A.R., Al-Fayez M.A., Al-Athari B.I., Al-Ajmi F.A., Al-Hazzaa H.M., Musaiger A.O., Ahmed F. (2014). Physical activity, sedentary behaviours and dietary habits among Kuwaiti adolescents: Gender differences. Public Health Nutr..

[58] Honkala S., Honkala E., Al-Sahli N. (2006). Consumption of sugar products and associated life-and school-satisfaction and self-esteem factors among schoolchildren in Kuwait. Acta Odontol. Scand..

[59] Al Muammar M., El Shafie M. (2014). Association between dietary habits and body mass index of adolescent females in intermediate schools in Riyadh, Saudi Arabia. East. Mediterr. Health J..

[60] Bello L., Al-Hammad N. (2006). Pattern of fluid consumption in a sample of Saudi Arabian adolescents aged 12–13 years. Int. J. Paediatr. Dent..

[61] Subih H.S., Abu-Shquier Y., Bawadi H., Al-Bayyari N. (2018). Assessment of body weight, maternal dietary knowledge and lifestyle practices among children and adolescents in north Jordan. Public Health Nutr..

[62] Institute of Medicine, Food and Nutrition Board Dietary Reference Intakes for Water, Potassium, Sodium, Chloride, and Sulfate. https://www.nal.usda.gov/sites/default/files/fnic_uploads/water_full_report.pdf.

[63] Jomaa L., Hwalla N., Constant F., Naja F., Nasreddine L. (2016). Water and beverage consumption among children aged 4–13 years in Lebanon: Findings from a National Cross-Sectional Study. Nutrients.

[64] Ali H.I., Al Dhaheri A.S., Elmi F., Ng S.W., Zaghloul S., Ohuma E.O., Qazaq H.S. (2019). Water and Beverage Consumption among a Nationally Representative Sample of Children and Adolescents in the United Arab Emirates. Nutrients.

[65] Al Sabbah H., Vereecken C., Kolsteren P., Abdeen Z., Maes L. (2007). Food habits and physical activity patterns among Palestinian adolescents: Findings from the national study of Palestinian schoolchildren (HBSC-WBG2004). Public Health Nutr..

[66] Kerkadi A., Sadig A.H., Bawadi H., Al Thani A.A.M., Al Chetachi W., Akram H., Al-Hazzaa H.M., Musaiger A.O. (2019). The relationship between lifestyle factors and obesity indices among adolescents in Qatar. Int. J. Environ. Res. Public Health.

[67] Al-Hazzaa H.M., Abahussain N.A., Al-Sobayel H.I., Qahwaji D.M., Musaiger A.O. (2011). Physical activity, sedentary behaviors and dietary habits among Saudi adolescents relative to age, gender and region. Int. J. Behav. Nutr. Phys. Act..

[68] Nazik M., Malde M., Ahmed M., Trovik T. (2013). Correlation between caries experience in Sudanese school children and dietary habits, according to a food frequency questionnaire and a modified 24-hr recall method. Afr. J. Food Agric. Nutr. Dev..

[69] FAO and FHI 360 (2016). Minimum Dietary Diversity for Women: A Guide for Measurement.

[70] Friel S., Hattersley L., Snowdon W., Thow A.M., Lobstein T., Sanders D., Barquera S., Mohan S., Hawkes C., Kelly B. (2013). Monitoring the impacts of trade agreements on food environments. Obes. Rev..

[71] El-Gazzar H.H., Saleh S.M., Khairy S.A., Marei A.S., ElKelany K., Al Soda M.F. (2019). Relationship between dietary intake and obesity among a group of primary school-aged children in Cairo Governorate. J. Med. Sci. Res..

[72] Khatib I., Hijazi S.S. (2009). Micronutrient deficiencies among children may be endemic in underprivileged areas in Jordan. Jordan Med. J..

[73] Al-Rewashdeh A. (2009). Assessment of the nutritional status for preschool children in Jordan. Bull. Fac. Agric. Cairo Univ..

[74] Institute of Medicine, Food and Nutrition Board Dietary Reference Intakes for Energy, Carbohydrate, Fiber, Fat, Fatty Acids, Cholesterol, Protein and Amino Acids. https://www.nap.edu/catalog/10490/dietary-reference-intakes-for-energy-carbohydrate-fiber-fat-fatty-acids-cholesterol-protein-and-amino-acids-macronutrients.

[75] Jabre P., Sikias P., Khater-Menassa B., Baddoura R., Awada H. (2005). Overweight children in Beirut: Prevalence estimates and characteristics. Child Care Health Dev..

[76] Akl C. (2012). Prevalence and Determinants of Overweight and Obesity in a Nationally Representative Sample of Lebanese Children 5 to 12 Years Old.

[77] Alredaisy M., Ibrahim S. (2011). Assessment of nutritional status of children less than 10 years old in rural western Kordafan. IIOABJ.

[78] Ng S.W., Zaghloul S., Ali H., Harrison G., Yeatts K., El Sadig M., Popkin B.M. (2011). Nutrition transition in the United Arab Emirates. Eur. J. Clin. Nutr..

[79] Kilani H., Al-Hazzaa H., Waly M.I., Musaiger A. (2013). Lifestyle Habits: Diet, physical activity and sleep duration among Omani adolescents. Sultan Qaboos Univ. Med. J..

[80] Bazhan M., Kalantari N., Houhiar-Rad A., Alavi-Majd H., Kalantari S. (2013). Dietary habits and nutrient intake in adolescent girls living in Northern Iran. Arch. Adv. Biosci..

[81] ALFaris N.A., Al-Tamimi J.Z., Al-Jobair M.O., Al-Shwaiyat N.M. (2015). Trends of fast food consumption among adolescent and young adult Saudi girls living in Riyadh. Food Nutr. Res..

[82] Washi S.A., Ageib M.B. (2010). Poor diet quality and food habits are related to impaired nutritional status in 13-to 18-year-old adolescents in Jeddah. Nutr. Res..

[83] Department of Health (1996). Dietary Reference Values for Food Energy, Nutrients for the United Kingdom. Report of the Panel on Dietary Values of the Committee on Medical Aspects of Food Policy.

[84] Zaghloul S., Al-Hooti S.N., Al-Hamad N., Al-Zenki S., Alomirah H., Alayan I., Al-Attar H., Al-Othman A., Al-Shami E., Al-Somaie M. (2013). Evidence for nutrition transition in Kuwait: Over-consumption of macronutrients and obesity. Public Health Nutr..

[85] Jildeh C., Papandreou C., Mourad T.A., Hatzis C., Kafatos A., Qasrawi R., Philalithis A., Abdeen Z. (2011). Assessing the nutritional status of Palestinian adolescents from East Jerusalem: A school-based study 2002–03. J. Trop. Pediatr..

[86] Ahmed N.M.K., Onsa Z.O. (2014). Nutritional Assessment of the Adolescents in the Northern State of Sudan. Pak. J. Nutr..

[87] Mohseni-Takalloo S., Mirmiran P., Hosseini-Esfahani F., Azizi F. (2014). Dietary fat intake and its relationship with serum lipid profiles in tehranian adolescents. J. Food Nutr. Res..

[88] World Health Organization (2008). Interim summary of conclusions and dietary recommendations on total fat & fatty acids. From the Joint FAO/WHO Expert Consultation on Fats and Fatty Acids in Human Nutrition.

[89] Nasreddine L., Chamieh M.C., Ayoub J., Hwalla N., Sibai A.-M., Naja F. (2020). Sex disparities in dietary intake across the lifespan: The case of Lebanon. Nutr. J..

[90] World Health Organization Diet, Nutrition and the Prevention of Chronic Diseases. https://apps.who.int/iris/bitstream/handle/10665/42665/WHO_TRS_916.pdf?sequence=1.

[91] Aounallah-Skhiri H., Traissac P., El Ati J., Eymard-Duvernay S., Landais E., Achour N., Delpeuch F., Romdhane H.B., Maire B. (2011). Nutrition transition among adolescents of a south-Mediterranean country: Dietary patterns, association with socio-economic factors, overweight and blood pressure. A cross-sectional study in Tunisia. Nutr. J..

[92] Huew R., Maguire A., Waterhouse P., Moynihan P. (2014). Nutrient intake and dietary patterns of relevance to dental health of 12-year-old Libyan children. Public Health Nutr..

[93] Montero P., Mora Urda A., Cherkaoui M., Anzid K. (2009). Transition nutritionnelle au Maroc: Étude comparative de l’état nutritionnel des adolescents entre 1991 et 2007. Bull Séances..

[94] Montero M.D.P., Mora-Urda A.I., Anzid K., Cherkaoui M., Marrodan M.D. (2017). Diet quality of Moroccan adolescents living in Morocco and in Spain. J. Biosoc. Sci..

[95] Anderson J.W., Baird P., Davis R.H., Ferreri S., Knudtson M., Koraym A., Waters V., Williams C.L. (2009). Health benefits of dietary fiber. Nutr. Rev..

[96] Collison K.S., Zaidi M.Z., Subhani S.N., Al-Rubeaan K., Shoukri M., Al-Mohanna F.A. (2010). Sugar-sweetened carbonated beverage consumption correlates with BMI, waist circumference, and poor dietary choices in school children. BMC Public Health.

[97] Tayel D.I., El-Sayed N.A., El-Sayed N.A. (2013). Dietary pattern and blood pressure levels of adolescents in Sohag, Egypt. J. Egypt Public Health Assoc..

[98] Al-Ansari J.M., Al-Jairan L.Y., Gillespie G.M. (2006). Dietary habits of the primary to secondary school population and implications for oral health. J. Allied Health.

[99] Rifat-uz-Zaman Z.I., Ali U. (2013). Dietary Intakes of Urban Adolescents of Sialkot, Pakistan Do Not Meet the Standards of Adequacy. Pak. J. Nutr..

[100] Aziz S., Hosain K. (2014). Carbohydrate (CHO), protein and fat intake of healthy Pakistani school children in a 24 hour period. J. Pak. Med. Assoc..

[101] Abuzaid O.I. (2012). Eating Patterns and Physical Activity Characteristics among Urban and Rural Students in Saudi Arabia.

[102] Institute of Medicine, Food and Nutrition Board Dietary Reference Intakes: RDA and AI for Vitamins and Elements. http://www.nationalacademies.org/hmd/~/media/Files/Activity%20Files/Nutrition/DRI-Tables/2_%20RDA%20and%20AI%20Values_Vitamin%20and%20Elements.pdf?la=en.

[103] Khatib I. (2002). High prevalence of subclinical vitamin A deficiency in Jordan: A forgotten risk. Food Nutr. Bull..

[104] Salamoun M., Kizirian A., Tannous R., Nabulsi M., Choucair M., Deeb M., Fuleihan G.E.-H. (2005). Low calcium and vitamin D intake in healthy children and adolescents and their correlates. Eur. J. Clin. Nutr..

[105] Al-Musharaf S., Al-Othman A., Al-Daghri N.M., Krishnaswamy S., Yusuf D.S., Alkharfy K.M., Al-Saleh Y., Al-Attas O.S., Alokail M.S., Moharram O. (2012). Vitamin D deficiency and calcium intake in reference to increased body mass index in children and adolescents. Eur. J. Pediatr..

[106] Saeid N., Elmzibri M., Hamrani A., Latifa Q., Belghiti H., El Berri H., Benjeddou K., Bouziani A., Benkirane H., Taboz Y. (2018). Assessment of sodium and potassium intakes in children aged 6 to 18 years by 24 h urinary excretion in city of rabat, Morocco. J. Nutr. Metab..

[107] Al-Daghri N.M., Al-Othman A., Alkharfy K.M., Alokail M.S., Khan N., Alfawaz H.A., Aiswaidan I.A., Chrousos G.P. (2012). Assessment of selected nutrients intake and adipocytokines profile among Saudi children and adults. Endocrine J..

[108] Nasreddine L.M., Kassis A.N., Ayoub J.J., Naja F.A., Hwalla N.C. (2018). Nutritional status and dietary intakes of children amid the nutrition transition: The case of the Eastern Mediterranean Region. Nutr. Res..

[109] Nasreddine L., Ayoub J.J., Al Jawaldeh A. (2018). Review of the nutrition situation in the Eastern Mediterranean Region. East. Mediterr. Health J..

[110] Kelishadi R., Ardalan G., Gheiratmand R., Majdzadeh R., Hosseini M., Gouya M., Razaghi E., Delavari A., Motaghian M., Barekati H. (2008). Thinness, overweight and obesity in a national sample of Iranian children and adolescents: CASPIAN Study. Child Care Health Dev..

[111] Mirmohammadi S.-J., Hafezi R., Mehrparvar A.H., Rezaeian B., Akbari H. (2011). Prevalence of overweight and obesity among Iranian school children in different ethnicities. Iran J. Pediatr..

[112] Nasreddine L., Naja F., Chamieh M.C., Adra N., Sibai A.-M., Hwalla N. (2012). Trends in overweight and obesity in Lebanon: Evidence from two national cross-sectional surveys (1997 and 2009). BMC Public Health.

[113] Bener A., Kamal A.A. (2005). Growth patterns of Qatari school children and adolescents aged 6–18 years. J. Health Popul. Nutr..

[114] Rootwelt C., Fosse K.B., Tuffaha A., Said H., Sandridge A., Janahi I., Greer W., Hedin L. Qatar s Youth Is Putting on Weight: The Increase in Obesity Between 2003 and 2009. Proceedings of the Qatar Foundation Annual Research Conference Proceedings.

[115] El-Hazmi M.A., Warsy A.S. (2002). A comparative study of prevalence of overweight and obesity in children in different provinces of Saudi Arabia. J. Trop. Pediatr..

[116] Al-Almaie S.M. (2005). Prevalence of obesity and overweight among Saudi adolescents in Eastern Saudi Arabia. Saudi Med. J..

[117] Al-Nuaim A.A., Al-Nakeeb Y., Lyons M., Al-Hazzaa H.M., Nevill A., Collins P., Duncan M.J. (2012). The prevalence of physical activity and sedentary behaviours relative to obesity among adolescents from Al-Ahsa, Saudi Arabia: Rural versus urban variations. J. Nutr. Metab..

[118] Aounallah-Skhiri H., El Ati J., Traissac P., Romdhane H.B., Eymard-Duvernay S., Delpeuch F., Achour N., Maire B. (2012). Blood pressure and associated factors in a North African adolescent population. a national cross-sectional study in Tunisia. BMC Public Health.

[119] Musaiger A.O. (2000). The state of nutrition in Bahrain. Nutr. Health.

[120] Musaiger A.O. (2011). Overweight and obesity in eastern mediterranean region: Prevalence and possible causes. J. Obes..

[121] Black R.E., Victora C.G., Walker S.P., Bhutta Z.A., Christian P., De Onis M., Ezzati M., Grantham-McGregor S., Katz J., Martorell R. (2013). Maternal and child undernutrition and overweight in low-income and middle-income countries. Lancet.

[122] Itoh H., Kanayama N. (2015). Nutritional conditions in early life and risk of non-communicable diseases (NCDs) from the perspective of preemptive medicine in perinatal care. Hypertens. Res. Pregnancy.

[123] Weiss R., Dziura J., Burgert T.S., Tamborlane W.V., Taksali S.E., Yeckel C.W., Allen K., Lopes M., Savoye M., Morrison J. (2004). Obesity and the metabolic syndrome in children and adolescents. N. Engl. J. Med..

[124] Khader Y., Batieha A., Jaddou H., El-Khateeb M., Ajlouni K. (2010). Metabolic syndrome and its individual components among Jordanian children and adolescents. Int. J. Pediatr. Endocrinol..

[125] Taha D., Ahmed O., Sadiq B.B. (2009). The prevalence of metabolic syndrome and cardiovascular risk factors in a group of obese Saudi children and adolescents: A hospital-based study. Ann. Saudi Med..

[126] Eapen V., Mabrouk A., Yousef S. (2010). Metabolic syndrome among the young obese in the United Arab Emirates. J. Trop. Pediatr..

[127] Ho K.K., Ferruzzi M.G., Wightman J.D. (2020). Potential health benefits of (poly) phenols derived from fruit and 100% fruit juice. Nutr. Rev..

[128] Tetens I., Alinia S. (2009). The role of fruit consumption in the prevention of obesity. J. Hortic. Sci. Biotech..

[129] Ello-Martin J.A., Roe L.S., Ledikwe J.H., Beach A.M., Rolls B.J. (2007). Dietary energy density in the treatment of obesity: A year-long trial comparing 2 weight-loss diets. Am. J. Clin. Nutr..

[130] Malik V.S., Schulze M.B., Hu F.B. (2006). Intake of sugar-sweetened beverages and weight gain: A systematic review^–^. Am. J. Clin. Nutr..

[131] Malik V.S., Pan A., Willett W.C., Hu F.B. (2013). Sugar-sweetened beverages and weight gain in children and adults: A systematic review and meta-analysis. Am. J. Clin. Nutr..

[132] Teff K.L., Grudziak J., Townsend R.R., Dunn T.N., Grant R.W., Adams S.H., Keim N.L., Cummings B.P., Stanhope K.L., Havel P.J. (2009). Endocrine and metabolic effects of consuming fructose-and glucose-sweetened beverages with meals in obese men and women: Influence of insulin resistance on plasma triglyceride responses. J. Clin. Endocrinol. Metab..

[133] Stanhope K.L., Schwarz J.M., Keim N.L., Griffen S.C., Bremer A.A., Graham J.L., Hatcher B., Cox C.L., Dyachenko A., Zhang W. (2009). Consuming fructose-sweetened, not glucose-sweetened, beverages increases visceral adiposity and lipids and decreases insulin sensitivity in overweight/obese humans. J. Clin. Investig..

[134] Stanhope K.L., Griffen S.C., Bair B.R., Swarbrick M.M., Keim N.L., Havel P.J. (2008). Twenty-four-hour endocrine and metabolic profiles following consumption of high-fructose corn syrup-, sucrose-, fructose-, and glucose-sweetened beverages with meals. Am. J. Clin. Nutr..

[135] Stanhope K.L., Havel P.J. (2008). Endocrine and metabolic effects of consuming beverages sweetened with fructose, glucose, sucrose, or high-fructose corn syrup. Am. J. Clin. Nutr..

[136] Muth N.D., Dietz W.H., Magge S.N., Johnson R.K., Pediatrics A.A.O., Association A.H. (2019). Public policies to reduce sugary drink consumption in children and adolescents. Pediatrics.

[137] Engin A. (2017). Fat cell and fatty acid turnover in obesity. Obes Lipotoxicity.

[138] Liberali R., Kupek E., Assis M.A.A.d. (2020). Dietary Patterns and Childhood Obesity Risk: A Systematic Review. Child Obes..

[139] Eyles H., Bhana N., Lee S.E., Grimes C., McLean R., Nowson C., Wall C. (2018). Measuring Children’s Sodium and Potassium Intakes in NZ: A Pilot Study. Nutrients.

[140] UNICEF-GAIN (2018). Food Systems for Children and Adolescents. Working Together to Secure Nutritious Diets.

[141] Al-Isa A. (2018). Nutritional Knowledge among High School Male Students in Kuwait. J. Community Med. Health Educ..

[142] Al-Yateem N., Rossiter R. (2017). Nutritional knowledge and habits of adolescents aged 9 to 13 years in Sharjah, United Arab Emirates: A crosssectional study. East. Mediterr. Health J..

[143] McClain A.D., Chappuis C., Nguyen-Rodriguez S.T., Yaroch A.L., Spruijt-Metz D. (2009). Psychosocial correlates of eating behavior in children and adolescents: A review. Int. J. Behav. Nutr. Phys. Act..

[144] Banna J.C., Buchthal O.V., Delormier T., Creed-Kanashiro H.M., Penny M.E. (2015). Influences on eating: A qualitative study of adolescents in a periurban area in Lima, Peru. BMC Public Health.

[145] Becker A.E., Burwell R.A., Herzog D.B., Hamburg P., Gilman S.E. (2002). Eating behaviours and attitudes following prolonged exposure to television among ethnic Fijian adolescent girls. Br. J. Psychiatry.

[146] Karimi-Shahanjarini A., Omidvar N., Bazargan M., Rashidian A., Majdzadeh R., Shojaeizadeh D. (2010). Iranian female adolescent’s views on unhealthy snacks consumption: A qualitative study. Iran J. Public Health.

[147] Anthrologica-World Food Programme (WFP) (2018). Bridging the Gap: Engaging Adolescents for Nutrition, Health and Sustainable Development.

[148] Pachón H., Simondon K.B., Fall S.T., Menon P., Ruel M.T., Hotz C., Creed-Kanashiro H., Arce B., Domínguez M.R.L., Frongillo E.A. (2007). Constraints on the delivery of animal-source foods to infants and young children: Case studies from five countries. Food Nutr. Bull..

[149] Armar-Klemesu M., Osei-Menya S., Zakariah-Akoto S., Tumilowicz A., Lee J., Hotz C. (2018). Using ethnography to identify barriers and facilitators to optimal Infant and Young Child Feeding in rural Ghana: Implications for programs. Food Nutr. Bull..

[150] Burns J., Emerson J.A., Amundson K., Doocy S., Caulfield L.E., Klemm R.D. (2016). A qualitative analysis of barriers and facilitators to optimal breastfeeding and complementary feeding practices in South Kivu, Democratic Republic of Congo. Food Nutr. Bull..

[151] Darmon N., Drewnowski A. (2015). Contribution of food prices and diet cost to socioeconomic disparities in diet quality and health: A systematic review and analysis. Nutr. Rev..

[152] Kelly B., Halford J.C., Boyland E.J., Chapman K., Bautista-Castaño I., Berg C., Caroli M., Cook B., Coutinho J.G., Effertz T. (2010). Television food advertising to children: A global perspective. Am. J. Public Health.

[153] Nasreddine L., Taktouk M., Dabbous M., Melki J. (2019). The extent, nature, and nutritional quality of foods advertised to children in Lebanon: The first study to use the WHO nutrient profile model for the Eastern Mediterranean Region. Food Nutr. Res..

[154] Amini M., Omidvar N., Yeatman H., Shariat-Jafari S., Eslami-Amirabadi M., Zahedirad M. (2014). Content analysis of food advertising in Iranian children’s television programs. Int. J. Prev. Med..

[155] World Health Organization Nutrition-Friendly Schools Initiative (NFSI). https://www.who.int/nutrition/topics/nutrition_friendly_schools_initiative/en/.

[156] World Health Organization (2017). Global Accelerated Action for the Health of Adolescents (AA-HA!): Guidance to Support Country Implementation.

[157] World Health Organization Regional Office for the Eastern Mediterranean (2018). Implementing the WHO Recommendations on the Marketing of Food and Nonalcoholic Beverages to Children in the Eastern Mediterranean Region.

[158] World Health Organization Regional Office for the Eastern Mediterranean (2019). Strategy on Nutrition for the Eastern Mediterranean Region 2020–2030.

[159] World Health Organization Regional Office for the Eastern Mediterranean (2019). Regional Framework for Action on Obesity Prevention 2019–2023.

[160] World Health Organization (2016). Policy Statement and Recommended Actions for Lowering Sugar Intake and Reducing Prevalence of Type 2 Diabetes and Obesity in the Eastern Mediterranean Region.

[161] World Health Organization (2014). Policy Statement and Recommended Actions for Reducing Fat Intake and Lowering Heart Attack Rates in the Eastern Mediterranean Region.

[162] World Health Organization (2014). Policy Statement and Recommended Actions to Lower National Salt Intakes and Lower Death Rates from High Blood Pressure and Strokes in the Eastern Mediterranean Region.

[163] HLPE (2017). Nutrition and food systems. A Report by the High Level Panel of Experts on Food Security and Nutrition of the Committee on World Food Security.

[164] UNICEF The Faces of Malnutrition. https://www.unicef.org/nutrition/index_faces-of-malnutrition.html.

